# Supplementary material for: A study on measuring the 222Rn in the Buriganga River and tap water of the megacity Dhaka
Source: PLoS One. 2023 May 23;18(5):e0286267. doi: 10.1371/journal.pone.0286267 (PMC10204947; doi:10.1371/journal.pone.0286267)
Supplement: Attachment — Submitted filename: PONE-D-22-28802_reviewer_comments.pdf [file pone.0286267.s001.pdf]

PLOS ONE

A pioneering study on measuring the 222Rn, a silent killer in the Buriganga River and tap water of the megacity Dhaka

--Manuscript Draft--

**Manuscript Number:**	PONE-D-22-28802
**Article Type:**	Research Article
**Full Title:**	A pioneering study on measuring the 222Rn, a silent killer in the Buriganga River and tap water of the megacity Dhaka
**Short Title:**	A pioneering study on measuring the 222Rn, a silent killer in the Buriganga River and tap water of the Dhaka
**Corresponding Author:**	Selina Yeasmin, Master of Science<br>Bangladesh Atomic Energy Commission<br>Dhaka, Shahbag BANGLADESH
**Keywords:**	Radon; RAD-H2O; Tap Water; River Water; Radiological Risks; Effective Dose
**Abstract:**	Radon, an inert gas, is considered a silent killer due to its carcinogenic characteristics. Dhaka city is situated on the banks of the Buriganga River, which is considered the lifeline of Dhaka city because it serves as a major source of the city's water supply for domestic and industrial purposes. As a first-time measurement of radon in the Buriganga River and tap water of Dhaka city, this study poses a great significance for the radiological safety of public health. A total of 30 water samples (10 tap water from Dhaka city and 20 surface samples from the Buriganga River) were collected and analyzed by using a RAD H2O detector for radon concentration. The average radon concentration in tap and river water was 1.537 ± 0.380 Bq/L and 0.675 ± 0.285Bq/L, respectively. All the values were found below the MCL (11.1 Bq/L) set by the USEPA, as well as the WHO-recommended safe limit of 100 Bq/L. The mean values of the total annual effective doses due to inhalation and ingestion were calculated to be 7.801 μSv/y and 3.426 μSv/y for tap water and river water, respectively. Although all these values were well below the permissible limit of 100 μSv/y proposed by WHO, they cannot be totally negligible because of the hazardous nature of radon, especially considering their entry to the human body via inhalation and ingestion pathways. The obtained data may serve as a reference for future radon-related works and contribute to develop the national safety limit for radon in water in Bangladesh.
**Order of Authors:**	M.S. Alam, Bachelor of Science<br>M.M. Mahfuz Siraz, Master of Science<br>Jubair A.M., Bachelor of Science<br>S.C. Das, Master of Science<br>D.A. Bradley, PhD<br>Mayeen Uddin Khandaker, PhD<br>Shinji Tokonami, PhD<br>Afroza Shelley, PhD<br>Selina Yeasmin, Master of Science
**Additional Information:**	
**Question**	**Response**
**Financial Disclosure**<br><br>Enter a financial disclosure statement that describes the sources of funding for the work included in this submission. Review the submission guidelines for detailed	The authors received no specific funding for this work.

Powered by Editorial Manager® and ProduXion Manager® from Aries Systems Corporation

requirements. View published research articles from *PLOS ONE* for specific examples.<br><br>This statement is required for submission and **will appear in the published article** if the submission is accepted. Please make sure it is accurate.<br><br>**Unfunded studies**<br>Enter: *The author(s) received no specific funding for this work.*<br><br>**Funded studies**<br>Enter a statement with the following details:<br>• Initials of the authors who received each award<br>• Grant numbers awarded to each author<br>• The full name of each funder<br>• URL of each funder website<br>• Did the sponsors or funders play any role in the study design, data collection and analysis, decision to publish, or preparation of the manuscript?<br>• **NO** - Include this sentence at the end of your statement: *The funders had no role in study design, data collection and analysis, decision to publish, or preparation of the manuscript.*<br>• **YES** - Specify the role(s) played.<br><br>* typeset	
**Competing Interests**<br><br>Use the instructions below to enter a competing interest statement for this submission. On behalf of all authors, disclose any competing interests that could be perceived to bias this work—acknowledging all financial support and any other relevant financial or non-financial competing interests.<br><br>This statement is required for submission and **will appear in the published article** if the submission is accepted. Please make sure it is accurate and that any funding sources listed in your Funding Information later in the submission form are also declared in your Financial Disclosure	The authors have declared that no competing interests exist.

Powered by Editorial Manager® and ProduXion Manager® from Aries Systems Corporation

statement.<br><br>View published research articles from *PLOS ONE* for specific examples.<br><br>**NO authors have competing interests**<br><br>Enter: *The authors have declared that no competing interests exist.*<br><br>**Authors with competing interests**<br><br>Enter competing interest details beginning with this statement:<br><br>*I have read the journal's policy and the authors of this manuscript have the following competing interests: [insert competing interests here]*<br><br>* typeset	
**Ethics Statement**<br><br>Enter an ethics statement for this submission. This statement is required if the study involved:<br><br>• Human participants<br>• Human specimens or tissue<br>• Vertebrate animals or cephalopods<br>• Vertebrate embryos or tissues<br>• Field research<br><br>Write "N/A" if the submission does not require an ethics statement.<br><br>General guidance is provided below. Consult the submission guidelines for detailed instructions. **Make sure that all information entered here is included in the Methods section of the manuscript.**	"N/A"

Powered by Editorial Manager® and ProduXion Manager® from Aries Systems Corporation

**Format for specific study types**<br><br>**Human Subject Research (involving human participants and/or tissue)**<br>• Give the name of the institutional review board or ethics committee that approved the study<br>• Include the approval number and/or a statement indicating approval of this research<br>• Indicate the form of consent obtained (written/oral) or the reason that consent was not obtained (e.g. the data were analyzed anonymously)<br><br>**Animal Research (involving vertebrate animals, embryos or tissues)**<br>• Provide the name of the Institutional Animal Care and Use Committee (IACUC) or other relevant ethics board that reviewed the study protocol, and indicate whether they approved this research or granted a formal waiver of ethical approval<br>• Include an approval number if one was obtained<br>• If the study involved *non-human primates*, add *additional details* about animal welfare and steps taken to ameliorate suffering<br>• If anesthesia, euthanasia, or any kind of animal sacrifice is part of the study, include briefly which substances and/or methods were applied<br><br>**Field Research**<br><br>Include the following details if this study involves the collection of plant, animal, or other materials from a natural setting:<br>• Field permit number<br>• Name of the institution or relevant body that granted permission	
**Data Availability**<br><br>Authors are required to make all data underlying the findings described fully available, without restriction, and from the time of publication. PLOS allows rare exceptions to address legal and ethical concerns. See the PLOS Data Policy and FAQ for detailed information.	Yes - all data are fully available without restriction

A Data Availability Statement describing where the data can be found is required at submission. Your answers to this question constitute the Data Availability Statement and **will be published in the article**, if accepted.<br><br>**Important:** Stating 'data available on request from the author' is not sufficient. If your data are only available upon request, select 'No' for the first question and explain your exceptional situation in the text box.<br><br>Do the authors confirm that all data underlying the findings described in their manuscript are fully available without restriction?	
**Describe where the data may be found in full sentences. If you are copying our sample text, replace any instances of XXX with the appropriate details.**<br><br>• If the data are **held or will be held in a public repository**, include URLs, accession numbers or DOIs. If this information will only be available after acceptance, indicate this by ticking the box below. For example: *All XXX files are available from the XXX database (accession number(s) XXX, XXX.).*<br>• If the data are all contained **within the manuscript and/or Supporting Information files**, enter the following: *All relevant data are within the manuscript and its Supporting Information files.*<br>• If neither of these applies but you are able to provide **details of access elsewhere**, with or without limitations, please do so. For example:<br><br>*Data cannot be shared publicly because of [XXX]. Data are available from the XXX Institutional Data Access / Ethics Committee (contact via XXX) for researchers who meet the criteria for access to confidential data.*<br><br>*The data underlying the results presented in the study are available from (include the name of the third party*	All relevant data are within the manuscript and its Supporting Information files.

Powered by Editorial Manager® and ProduXion Manager® from Aries Systems Corporation

and contact information or URL).<br>• This text is appropriate if the data are owned by a third party and authors do not have permission to share the data.<br><br>* typeset	
Additional data availability information:	

Manuscript
Click here to access/download;Manuscript;Plos One Radon Paper 17-10-22.docx

A pioneering study on measuring the ^{222}Rn, a silent killer in the Buriganga River and tap water of the megacity Dhaka

M.S. Alam[a], M.M. Mahfuz Siraz[b], Jubair A.M.[a], S.C. Das[c], D.A. Bradley[d,e], Mayeen Uddin Khandaker[e,f], Shinji Tokonami[g], Afroza Shelley[a] and S.Yeasmin[b]*

[a]Department of Nuclear Engineering, University of Dhaka, Dhaka-1000, Bangladesh

[b]Health Physics Division, Atomic Energy Centre, Dhaka-1000, Bangladesh

[c]Institute of Nuclear Minerals, Bangladesh Atomic Energy Commission, Savar, Dhaka 1207, Bangladesh

[d]Centre for Nuclear and Radiation Physics, Department of Physics, University of Surrey, Guildford, Surrey GU2 7XH, UK

[e]Centre for Applied Physics and Radiation Technologies, School of Engineering and Technology, Sunway University, 47500, Bandar Sunway, Selangor, Malaysia

[f]Department of General Educational Development, Faculty of Science and Information Technology, Daffodil International University, DIU Rd, Dhaka, 1341, Bangladesh

[g]Institute of Radiation Emergency Medicine, Hirosaki University, 66-1 Honcho, Hirosaki 036-8564, Japan

*Corresponding author email: selinayeasmin@yahoo.com

Abstract

Radon, an inert gas, is considered a silent killer due to its carcinogenic characteristics. Dhaka city is situated on the banks of the Buriganga River, which is considered the lifeline of Dhaka city because it serves as a major source of the city's water supply for domestic and industrial purposes. As a first-time measurement of radon in the Buriganga River and tap water of Dhaka city, this study poses a great significance for the radiological safety of public health. A total of 30 water samples (10 tap water from Dhaka city and 20 surface samples from the Buriganga River) were collected and analyzed by using a RAD H_2O detector for radon concentration. The average radon concentration in tap and river water was 1.537 ± 0.380 Bq/L and 0.675 ± 0.285Bq/L, respectively. All the values were found below the MCL (11.1 Bq/L) set by the USEPA, as well as the WHO-recommended safe limit of 100 Bq/L. The mean values of the total annual effective doses due to inhalation and ingestion were calculated to be 7.801 µSv/y and 3.426 µSv/y for tap water and river water, respectively. Although all these values were well below the permissible limit of 100 µSv/y proposed by WHO, they cannot be totally negligible because of the hazardous nature of radon, especially considering their entry to the

human body via inhalation and ingestion pathways. The obtained data may serve as a reference for future radon-related works and contribute to develop the national safety limit for radon in water in Bangladesh.

Keywords: Radon, RAD-H_2O, Tap Water, River Water, Radiological Risks, Effective Dose

1. Introduction

Human beings are continuously exposed to natural radiation, mostly coming from terrestrial and extra-terrestrial sources (Rani et al., 2021). Among the existing sources of ionizing radiation in the environment, Radon (^{222}Rn) alone is the major contributor (more than 50%) of the total radiation dose to humans (UNSCEAR, 2010). ^{222}Rn is the only gaseous element in the ^{238}U decay series and possesses no color, odor, or taste. This (^{222}Rn) short-lived ($T_{1/2}$=3.82 days) radioactive nucleus is formed due to the alpha decay of ^{226}Ra. Among the three naturally occurring radioisotopes of radon, ^{222}Rn is the most abundant in nature as Thoron (^{220}Rn) and Actinon (^{219}Rn) have relatively very short half-lives of 55s and 3.2s, respectively.

Radon is present naturally in the earth's strata. Its abundance in the earth's crust fluctuates with the variation of geology and lithology of the area. Due to its high mobility, radon gas can swiftly travel from soil and rocks to water and air. Albeit, the concentration of radon in water depends on the temperature, lithology, geology, rainfall, and earthquake activities (Faweya et al., 2021). ^{222}Rn is highly volatile, easily dissolved, and emanates from the water. Since safe water is essential for human lives, it is a matter of great concern to evaluate the radon level in the water sources such as rivers and tap water. A relatively higher concentration of radon is found in groundwater than in surface water due to the aeration process (Faweya et al., 2021). Because of its gaseous nature, ^{222}Rn is used as a tectonic tracer (Ahmad et al., 2019) to determine the tectonic fault lines and predict earthquakes.

Radon is considered a hazardous gas due to its potential to affect human cells and tissues biologically. Ingestion through the gastrointestinal tract and inhalation via the respiratory tract are the two major pathways of entering radon into the human body. Both pathways are potentially risky, affecting the lung and the gastrointestinal system. Although radon is chemically inactive with other substances and biological cells, it is the second most prominent cause of lung cancer after tobacco smoking (WHO 2009). In the case of inhalation, the short-lived metallic progeny of radon (mostly ^{218}Po and ^{214}Po) are deposited in the lungs

and damage the cells and the tissues of the respiratory system via high-energy alpha emission. That is why it is one of the main contributors to escalating lung cancer risks. The IARC (The International Agency for Research on Cancer), a part of WHO, classified radon as a group 1 carcinogen (Copes & Peterson, 2014; Ismail et al., 2021a).

Water is vital for all life; human beings use water regularly for various purposes, including bathing, drinking, etc. However, water consumption is the primary cause of radon exposure through the ingestion pathway, whereas the emanation of radon from water causes exposure through the inhalation of air. As radon is loosely soluble in water, it can easily emanate from water to air (Thumvijit et al., 2020a). For that reason, radon activity measurement in water is necessary to protect people from radiological hazards. Many international organizations propose a safe limit on radon concentration in water, and almost all developed countries have their national guidelines for radiation safety. The World Health Organization recommended a safe limit of 100 Bq/L for radon in the water (WHO, 2008a), whereas the USEPA suggested the maximum contamination level (MCL) of 11.1 Bq/L (US EPA, 2012). The USEPA also proposes an alternative maximum contamination level (AMCL) of 148 Bq/L (US EPA, 2012). To apprehend the health hazard of radon, measurement of the annual effective dose due to radon ingestion and inhalation is essential. The WHO and IAEA recommend that the total annual effective dose due to radon in water should be < 100 μSv(WHO, 2017).

Numerous studies have been performed worldwide to measure the radon level in various water resources such as tap water, river water, deep well water, bore well water, bottled water, etc.(Faweya et al., 2021; Mustapha et al., 2002; Rani et al., 2021; Thumvijit et al., 2020a; Yong et al., 2021). Almost every developed country has its national reference limit of radon in water and indoor air to ensure radiological safety for public health. Bangladesh has no such reference level for radon in water. In fact, to the best of our knowledge, no studies on radon measurement in surface water, tap water, or indoor air have yet been conducted. Millions of people living in the Dhaka megacity solely rely on tap water for their daily household purposes, such as washing, bathing, drinking, cooking, etc. Recent studies claim that tap water contains excessive heavy metals(Hossain et al., n.d.). The Buriganga river has already been heavily polluted, and recent studies show that the situation is getting worse day by day(Bhuiyan et al., 2015; Whitehead et al., 2019). Nevertheless, it meets around 20% of the total water demand of Dhaka city dwellers. In addition, it serves as one of the busiest major transportation routes/hubs, as well as many businesses and trade centers that are situated on the bank of this

river. This indicates a greater possibility of radon exposure to the general populace. So, it is necessary to measure the radon level in the tap water and the Buriganga river water to find out if it is within the safe limit or not, which eventually will help to ensure the radiological safety of public health.

The purpose of this study is to (a) measure radon concentration in the chemically and biologically polluted Buriganga river water and the tap water of the megacity Dhaka, b) calculate the associated radiological hazards, c) to contribute to the setting up of a factual baseline data which will assist the authority to structure a national reference level of radon water.

2. Methodology

2.1 Study Area

Dhaka, the capital city of Bangladesh, as well as one of the most densely populated megacities in the world, is the prime focus of this study. Dhaka is located between latitudes 23°42' and 23°54'N and longitudes 90°20' and 90°28'E. The geographical area of this city is 306.38 square kilometers, where more than 21 million people inhabit (Bangladesh Bureau of Statistics, 2015). Several rivers like Buriganga, Balu, Tongi Khal, and Turag surround the city from the south, east, west, and north (*Dhaka, Geology - Banglapedia*, n.d.), respectively. However, the Buriganga river has a major share, and it forms the southern and western boundaries of Dhaka city. The length of this river flowing through Dhaka is 11 km, the depth is 10m, and the width is 400m. The latitude and the longitude of this river are 23° 37' 59.99" N, 90° 25' 59.99" E (*Buriganga Riverkeeper : » History*, n.d.). Because of the large-scale industrial activities on the bank of the Buriganga river, it has become the worst polluted river in the country.

2.2 Geology of Dhaka city and its periphery region

The megacity Dhaka is placed at the southern end of the Madhupur tract, 1.5–10 m (average 6 m) above the adjoining floodplains (Burgess et al., 2011). The area is characterized by Quaternary alluvial sequences of the Madhupur Tract, known as Pleistocene terrace deposits surrounding Holocene deposits of the peripheral rivers (Bodrud-Doza et al., 2020; Mohammad

Abdul Hoque, 2004; Rahman et al., 2013). The geological map and the cross section along NS and EW of the study area are illustrated in Fig. 1(b) and Fig. 1(d), showing different geological units present in this area. The Pleistocene terrace deposits of varying thickness (an average of 10 m thick in Dhaka) are subdivided by Upper and Lower Madhupur Clay deposits. The Upper Madhupur Clay deposits are reddish brown to pale yellow sticky clay and silty clay, containing ferruginous nodules and dark spots of manganese, compacted highly weathered and oxidized residual deposits.

On the other hand, the Lower Madhupur Clay deposits primarily contain pale yellowish to yellowish brown sandy clay to clayey sand and silty sand with similar nodules and spots but less weathered and oxidized than the upper (Burgess et al., 2011; Karim et al., 2019). The Holocene deposits are further subdivided into alluvial flood plain deposits comprising natural levee deposits, bar deposits, point bar deposits, back swamp deposits, flood plain deposits, and valley fill deposits. Flood plain deposits mainly comprise grey to dark grey colour sticky clay to clayey silt, with discontinuous sand, oxidized root, rootlets, and organic matter. The valley fill deposits consist of dark grey to yellowish to olive brown, silty clay, marshy clay, and peat (Karim et al., 2019). A sequence of fine to coarse-grained micaceous quartzofeldspathic sands containing Dupi Tila Formation of Pliocene age, hydro geologically known as the Dupi Tila aquifers, the primary aquifer of Dhaka city, underlies the Madhupur Clay and is not exposed anywhere in the city (Bodrud-Doza et al., 2020; Burgess et al., 2011; DWASA, 2008; Rahman et al., 2013). A gravel bed lies at the bottom of the Dupi Tila Formation, which grades upward from coarse-grained sands to medium-grained sands to fine-grained sands at the top. The Dupi Tila Formation is divided by a discontinuous clay layer into two aquifers: an upper fine-grained aquifer (approximately 40–50 m thick) and a lower coarse-grained aquifer (approximately 80 m thick) (Burgess et al., 2011). A summary of the Pliocene to Recent lithological and aquifer characteristics of the study area is depicted in Fig. 1(c). The geochemical study of the

groundwater of Dupi Tila aquifer shows that the Ca/Mg-HCO3 type and weathering of aluminosilicates control the distribution of major ions in the aquifers (Islam et al., 2021). The Dupi Tila and Madhupur Formations are isolated by extensive incision of the land surface during the late Quaternary and forming several faults at their boundaries which affect the aquifer river system and the groundwater flow of this area (Bodrud-Doza et al., 2020; M Kamrul Hasan et al., 1999; Mohammad A Hoque et al., 2007; Rahman et al., 2013). It is assumed that due to the elevation of the river bed with the top of the Dupi Tila sands has through the connection between them and the rivers surrounding Dhaka (i.e., Buriganga, Balu, and Turag River) and the aquifer is possible along certain reaches (Ahmed & Burgess, 2003; Burgess et al., 2011; M K Hasan et al., 1998).

Fig 1. (a) The study area map showing the sampling location of river water (RW) and tap water (TW). (b) The geology of the study area. (c) Lithostratigraphy of the study area (Burgess et al., 2011). (d) Cross-section along the NS and EW direction in the study area.

2.3 Sampling:

Thirty water samples, including 20 river water and 10 tap water (Fig. 1a), were collected in November 2021 using a 500 mL plastic bottle prior to the winter season. The river water samples were collected from the highly polluted Buriganga river by following the stratified sampling technique approved by IAEA (International Atomic Energy Agency, 2019). The majority of the samples were collected from heavily populated river bank areas such as Sadarghat, Showari Ghat, Mitford ghat, Gabtoli, etc. The bottle was fully submerged directly into the water during the river water collection to prevent air bubbles in the bottle. The tap waters were collected from different localities of the megacity Dhaka using a systematic grid sampling technique approved by the IAEA (International Atomic Energy Agency, 2019). Before sample collection, the tap was opened for several minutes, and the water was allowed to flow. Afterward, the bottle was filled and sealed tightly. Prevention of aeration during sampling was the prime concern to avoid the escape of dissolved radon in the water. Each of the samples was labeled with a unique sample ID (RW for river water and TW for tap water), and the GPS of the collection points and the collection time were recorded. These water

samples were taken immediately to the Laboratory of the Health Physics Division in Atomic Energy Centre Dhaka of Bangladesh Atomic Energy Commission.

2.4 Experimental Procedure:

The radon activity concentration in collected water samples was measured using RAD7, a portable electric radon detector with RAD-H_2O accessories (manufactured by Durridge Co. Ltd). A schematic diagram of the experimental setup is illustrated in Fig. 2. The inner cell of the RAD7 is a hemisphere coated with an electrical conductor where the energy of emitted alpha particles from radon and its progeny are converted into electrical signals. Before analyzing the samples, the RAD7 detector needs to be radon free and dry. Dry air was purged for 10 minutes, lowering the humidity below 10%. The collected water samples were transferred into a 250 mL glass vial and connected with the RAD7. The radon emanation occurred by aerating the water via a glass frit in a closed-loop system. An internal air circulating pump recirculates the air through the closed-loop system to extract the radon from the water until the equilibrium is reached. The wat-250 process was selected to measure radon in water, where the extraction efficiency was 94%. The equilibrium state is reached within 5 minutes, and after this, no more radon can be extracted from the water. The air is circulated by the pump aerating the water and supplying the radon to the RAD7 detector. This process runs for 30 minutes in four cycles to measure the radon in the samples. The RAD7 summarizes the average and corrected radon concentration measurements obtained from each sample for four cycles at the end of the run in a printout.

Fig 2. Schematic diagram of RAD-H_2O detector closed loop aeration system, where the air and water volume remain constant and independent of the flow rate.

2.5 Dosimetry Calculation

Internal radon exposure comes primarily from radon inhalation and ingestion, which is harmful to the respiratory organs. When water is collected and used, radon is inhaled, and radon is ingested when radon-contaminated water is consumed. Therefore, by using Equations (1) and (2), the annual effective dose due to radon inhalation and ingestion is calculated from the

experimentally measured values of the radon concentration (Ismail et al., 2021a; Rani et al., 2021; Thumvijit et al., 2020b; UNSCEAR, 2010).

$$\sum D_{ig}\ (\mu Sv/y) = C_{RnW} \times C_W \times EDC \times 10^{-3} \quad \dots\dots\dots\dots\dots\dots\dots\dots\dots..(1)$$

$$\sum D_{in}\ (\mu Sv/y) = C_{RnW} \times R_{AW} \times F \times O \times DCF \times 10^{-3} \quad \dots\dots\dots\dots(2)$$

Where,

$\sum D_{ig}$ and $\sum D_{in}$ represents effective doses due to ingestion and inhalation, respectively

C_{RnW} = Radon activity concentration in collected samples measured by RAD-H_2O detector (Bq/L)

C_W = Water consumption per year (730 L/day) (Thumvijit et al., 2020a)

EDC (Effective Dose Coefficient) = 3.5 nSv/Bq for radon ingestion (UNSCEAR, 2010)

10^{-3} is used for the conversion of nano-to-micro

R_{AW} = ratio of radon in the air to water (10^{-4}) (UNSCEAR, 2010)

F (equilibrium factor between radon and its progeny) = 0.4 (UNSCEAR, 2010)

O (mean indoor occupancy factor) = 7000 h/y (UNSCEAR, 2010)

DCF (dose conversion factor for radon exposure) = 9 nSv/h (Bq/m^3) (UNSCEAR, 2010)

3. Results and Discussion

3.1 Radon in river water:

As demonstrated in Table 1, the measured radon concentration in the collected twenty river water samples from the highly polluted Buriganga river varied from 0.349 ± 0.180 to 1.160 ± 0.610 Bq/L with an average of 0.675 ± 0.285Bq/L. The maximum radon concentration (1.160 ± 0.610 Bq/L) was found in the sample collected from the Forashgonj Kheyaghat area (RW17). There is a direct swage-drain line (from the Dolai Khal) near the Forashgonj Kheyaghat, which may contaminate the area with technologically enhanced naturally occurring radioactive materials (TENORMs), consequently may increase the radon level in that location. The sample collected from the Wais Ghat area (RW15) had the minimum radon concentration

(0.349 ± 0.180 Bq/L). The radon level in these river water samples is relatively low as the aeration of surface water accelerates the emanation of radon into the environment (Ismail et al., 2021b; Orosun et al., 2021). No sample either contained a radon concentration level more than the safe limit of 100 Bq/L recommended by the WHO or exceeded the maximum contamination limit (MCL) of 11.1 Bq/L set by USEPA (Al Zabadi et al., 2012a; Şahin Bal et al., 2021; States, 1999; Yong et al., 2021).

For each river water sample, the annual dose due to radon inhalation and ingestion is listed in Table 1. The mean annual effective dose due to river water ingestion and inhalation were 1.725 µSv/y and 1.701 µSv/y, respectively. The total annual effective dose for river water ranged from 1.771 µSv/y to 5.887 µSv/y with an average of 3.426 µSv/y. All of these values were well below the maximum permissible limit of 100 µSv/y set by WHO (Darabi Fard et al., 2020; Ismail et al., 2021a; Kareem et al., 2020; *WHO*, 2008b).

Table 1: Measured radon concentration and calculated effective doses for the Buriganga river water

**Sample ID**	**Location Near**	**Latitude**	**Longitude**	**Mean Concentration (Bq/L)**	**Annual Effective Dose of Ingestion *(µSv/y)***	**Annual Effective Dose of Inhalation *(µSv/y)***	**Total Annual Effective Dose *(µSv/y)***
**RW01**	Aminbazar Bridge	23.7843125	90.3362020	0.629 ± 0.268	1.607	1.585	3.192
**RW02**	Gabtoli Balughat 2	23.7802673	90.3374692	0.664 ± 0.135	1.697	1.673	3.370
**RW03**	Gabtoli Balughat 1	23.7789416	90.3385024	0.699 ± 0.410	1.786	1.761	3.547
**RW04**	Azim Tower	23.7687041	90.3442110	0.908 ± 0.489	2.320	2.288	4.608
**RW05**	Basila Bridge	23.7442182	90.3466333	0.629 ± 0.141	1.607	1.585	3.192
**RW06**	Jhauchar Ghat	23.7336485	90.3561759	0.559 ± 0.361	1.428	1.409	2.837
**RW07**	Gudara Ghat	23.7145463	90.3612411	0.524 ± 0.310	1.339	1.320	2.659
**RW08**	Jadbar Bazar Ghat	23.7085396	90.3786198	0.525 ± 0.240	1.341	1.323	2.664
**RW09**	Showari Ghat	23.7113611	90.3947077	0.734 ± 0.177	1.875	1.850	3.725
**RW10**	Imamgonj Ghat	23.7113133	90.3965892	0.490 ± 0.181	1.252	1.235	2.487
**RW11**	Mitford Ghat	23.7109461	90.3994460	0.943 ± 0.499	2.409	2.376	4.786
**RW12**	Mitford Hospital Ghat	23.7109609	90.3994378	0.455 ± 0.070	1.163	1.147	2.309
**RW13**	Babu Bazar Terminal	23.7095952	90.4025324	0.524 ± 0.384	1.339	1.320	2.659
**RW14**	Badamtoli Ghat	23.7082794	90.4051203	0.909 ± 0.332	2.323	2.291	4.613
**RW15**	Wais Ghat	23.7066571	90.4078869	0.349 ± 0.180	0.892	0.879	1.771
**RW16**	Sadarghat	23.7045832	90.4114031	0.420 ± 0.115	1.073	1.058	2.132
**RW17**	Forashgonj Kheyaghat	23.7002854	90.4167060	1.160 ± 0.610	2.964	2.923	5.887

**RW18**	Dhaka Saw Mill	23.6937433	90.4228607	0.805 ± 0.211	2.057	2.029	4.085
**RW19**	Postogola Bridge	23.6898054	90.4263290	0.804 ± 0.209	2.054	2.026	4.080
**RW20**	Shyampur fire service	23.6835213	90.4344459	0.770 ± 0.371	1.967	1.940	3.908
**Average**				**0.675 ± 0.285**	**1.725**	**1.701**	**3.426**
**Minimum**				**0.349 ± 0.180**	**0.892**	**0.879**	**1.771**
**Maximum**				**1.160 ± 0.610**	**2.964**	**2.923**	**5.887**

In Table 2, the present study for river water is compared with the reported results worldwide. The radon level was found very high in some river water, such as the radon level (60 Bq/L) in the Rajouri of Pir Panjal, Kashmir was high due to the mountainous area where many minerals were found in the soil of that region (Nazir et al., 2020). The study at Ekiti, Nigeria, claimed that the high radon level (42-88 Bq/L) was found in river water due to the local geology covered with migmatite, porphyritic granite, granite gneiss, and undifferentiated schist (Faweya et al., 2021). In another study, the authors claimed the Gold and Bismuth mining site near the study area in Edu LGA, Kwara State, Nigeria, was the main reason for the high radon level (19.14 ± 3.98 Bq/L)(Lawal, 2021). Nevertheless, the geological map of the Buriganga shows that there are no mountains or volcanic areas around this river. Neither any mining site nor the study area was covered with minerals. The Buriganga riverbed is mainly clay instead of rocks (*Dhaka, Geology - Banglapedia*, n.d.). These were the significant reasons for the low radon level in this river water. Additionally, the result of this study is consistent with the previous research carried out in different regions of the world, such as in India (Rajashekara et al., 2007; Shivanandappa & Yerol, 2018), Iraq(Kareem et al., 2020), and Malaysia (Ismail et al., 2021b).

Table 2: A worldwide comparative scenario of the radon level in river water

Country/ Region	Mean Radon Concentration (Bq/L)	Reference
Peninsular, Malaysia	5.04	(Ismail et al., 2021b)
Kwara,Nigeria	15.97	(Orosun et al., 2021)
Ekiti, Nigeria	42.22 to 88.22	(Faweya et al., 2021)
Edu, Nigeria	19.14 ± 3.98	(Lawal, 2021)
Punjab, India	3.37 ± 0.29	(Rani et al., 2021)
Rajouri, Pir Panjal	60	(Nazir et al., 2020)
Kirkuk, Iraq	0.359	(Kareem et al., 2020)
Hemavathi River, India	0.67	(Shivanandappa & Yerol, 2018)
Transylvania, Romania	0.9 to 4.5	(Nita et al., 2013)
Karnataka, India	0.16 to 1.79	(Rajashekara et al., 2007)
Dhaka, Bangladesh	**0.675 ± 0.285**	Present work

3.2 Radon in tap water:

As illustrated in Table 3, the radon concentration in the ten tap water samples collected from Dhaka city varied from 0.559 ± 0.302 to 3.060 ± 0.605 Bq/L with an average of 1.537 ± 0.380 Bq/L. The lowest radon concentration (0.559 ± 0.302 Bq/L) was found in the Bongshal area (TW10). The sample collected from the Mohammadpur area (TW04) contained the highest radon concentration (3.060 ± 0.605 Bq/L). A thorough investigation found that deep tube well water was stored in a tank and then supplied to the tap in the house from where the TW04 was collected. The water was stored in a closed tank that prevented air contact with water. For this reason, the radon gas hardly emanates from the water, so the radon level was higher than the others. However, all the samples contained lower radon levels than both the maximum contamination limit (MCL) of 11.1 Bq/L set by USEPA and the safe limit of 100 Bq/L recommended by the WHO (Al Zabadi et al., 2012a; Şahin Bal et al., 2021; States, 1999; Yong et al., 2021).

The annual effective dose due to radon inhalation and ingestion for each tap water sample is listed in Table 3. The maximum and the minimum values of annual effective dose due to tap water ingestion were 7.818 µSv/y and 1.428 µSv/y, with an average of 3.928 µSv/y. For inhalation, it ranged from 1.409 µSv/y to 7.711µSv/y with a mean of 3.874 µSv/y. The total annual effective dose for tap water ranged from 15.530 µSv/y and 2.837 µSv/y with an average of 3.932 µSv/y. All of these values were way below the maximum permissible limit of 100 µSv/y set by WHO (Darabi Fard et al., 2020; Ismail et al., 2021a; Kareem et al., 2020; *WHO*, 2008b).

Table 3: Measured radon concentration and calculated effective doses for the tap water

**Sample ID**	**Location Near**	**Latitude**	**Longitude**	**Mean Concentration (Bq/L)**	**Annual Effective Dose of Ingestion *(µSv/y)***	**Annual Effective Dose of Inhalation *(µSv/y)***	**Total Annual Effective Dose *(µSv/y)***
**TW01**	Rupnagar	23.8140410	90.3542840	2.450 ± 0.711	6.260	6.174	12.434
**TW02**	Mirpur 11	23.8187101	90.3736211	1.640 ± 0.321	4.190	4.133	8.323
**TW03**	Shyamoli	23.7738063	90.3641953	0.909 ± 0.179	2.322	2.291	4.613
**TW04**	Mohammadpur	23.7537414	90.3607778	3.060 ± 0.605	7.818	7.711	15.530
**TW05**	Farmgate	23.7570560	90.3898441	1.190 ± 0.354	3.040	2.999	6.039

**TW06**	Baridhara	23.7991200	90.4240744	2.590 ± 0.479	6.617	6.527	13.144
**TW07**	Rampura	23.7648065	90.4217147	0.629 ± 0.080	1.607	1.585	3.192
**TW08**	Kamalapur	23.7321814	90.4264804	1.610 ± 0.369	4.114	4.057	8.171
**TW09**	Gulistan	23.7272202	90.4126653	0.735 ± 0.403	1.878	1.852	3.730
**TW10**	Bongshal	23.7215800	90.4057534	0.559 ± 0.302	1.428	1.409	2.837
**Average**				**1.537 ± 0.380**	**3.928**	**3.874**	**7.801**
**Maximum**				**3.060 ± 0.605**	**7.818**	**7.711**	**15.530**
**Minimum**				**0.559 ± 0.302**	**1.428**	**1.409**	**2.837**

Table 4 compares the present study for tap water with the reported literature worldwide. According to the previous literature, high radon level in tap water was found in some countries. A study in the Sabzevaran fault, Iran, found the radon level in tap water higher (17.12 Bq/L) than in the MCL. The authors concluded that a high radon level was due to volcanic, metamorphic, and sedimentary rocks surrounding the study area (Shamsaddini et al., 2020). A study in Kenya found the radon level (37 Bq/L) much higher than the MCL in tap water samples; due to the studied area being located near a volcanic region, and the maximum tap water of the area was collected from underground water sources, the local geology was the primary reason for the abnormally higher radon level (Mustapha et al., 2002).

Table 4: A worldwide comparative scenario of the radon level in tap water

Country/ Region	Mean Radon Concentration (Bq/L)	Reference
Penang, Malaysia	0.066	(Salih, 2021)
Bitlis, Turkey	0.59 to 66.00	(Şahin Bal et al., 2021)
Xinjiang, China	0.543	(Yong et al., 2021)
Chiang Mai, Thailand	0.18 - 1.13	(Thumvijit et al., 2020a)
Sabzevaran fault, Iran	17.12	(Shamsaddini et al., 2020)
Zarand, Iran	5.16 to 14.4	(Darabi Fard et al., 2020)
Kabini River Basin, India	8.5	(Yashaswini et al., 2020)
Sik, Malaysia	0.0171 ± 0.0036	(Ahmad et al., 2019)
Giresun University, Turkey	0.98 to 27.28	(Öge & Gökçe, 2018)

Rajasthan, India	0.5 to 15	(Mittal et al., 2016)
Kedah, Malaysia	7.0 ± 0.71	(Ahmad et al., 2015)
Bihor, Romania	6.9	(Moldovan et al., 2014)
Nablus, Palestine	1.0	(Al Zabadi et al., 2012b)
Bursa, Turkey	0.91 to 12.58	(Akar Tarim et al., 2012)
Kenya	37	(Mustapha et al., 2002)
Dhaka, Bangladesh	**1.537 ± 0.380**	Present work

The tap water of Dhaka city is collected from surface water treatment plants as well as extracted the underground water by using different pumps (*Urban Water Blueprint - Dhaka*, n.d.), which are then supplied all over the city through a piping system. However, the geology of the present study area neither consisted of any volcanic, granitic, or metamorphic rock nor any volcanic region nearby. These may be the leading causes of the lower radon level in the tap water of Dhaka city. Moreover, the result of this study is consistent with many studies conducted in China (Yong et al., 2021), Thailand (Thumvijit et al., 2020a), Palestine (Al Zabadi et al., 2012b), Malaysia (Salih, 2021), India (Yashaswini et al., 2020).

The present study shows that the radon level in river water is much lower than in tap water. River water is easily in contact with the open air, which accelerates the emanation of radon, while tap water has less contact with the air. Tap water is supplied in a closed piping system from the storage tank to the tap, and so the aeration is negligible compared to surface water. Additionally, a portion of the tap water of Dhaka city is supplied from a groundwater source which was the primary reason for the high radon level in some samples like TW04.

According to the Stochastic model of radiation, there is no threshold limit; even a single atom can cause severe damage to the body, so a slight amount of radon in water should be a concern. Even though all of the measured values of the radon level in the water and the associated effective dose were below the USEPA and the WHO limit, a radon mitigation treatment plant should be introduced. The water should be aerated and boiled before consumption, reducing the radon concentration in the water. No national reference level has yet been set for radon in water, so this study being the first radon-related work in Dhaka city, may contribute to forming a national safety limit for radon in water.

4. Conclusion

In a pioneering study in Dhaka city, radon level in river water and tap water was measured using a RAD H_2O detector to ensure public health safety from radiological hazards. The ranges of measured radon concentrations in the river water (0.349 ± 0.180 to 1.160 ± 0.610 Bq/L) and the tap water (0.559 ± 0.302 to 3.060 ± 0.605 Bq/L) showed lower than the limit set by the WHO and the USEPA (States, 1999). Also, the total annual effective doses were within the safe limit set by the WHO (*WHO*, 2008b).

Considering the carcinogenic characteristics of ^{222}Rn, a radon mitigation program should be introduced, and frequent monitoring of radon in various dwelling media is essential to ensure the safety of public health. Further extensive research should be carried out for radon mapping of the country. Moreover, this study may serve as factual reference data to set a national reference radon limit which is currently absent in the country.

References

Ahmad, N., Jaafar, M. S., & Alsaffar, M. S. (2015). Study of radon concentration and toxic elements indrinking and irrigated water and its implications in Sungai Petani, Kedah, Malaysia. *Journal of Radiation Research and Applied Sciences*, *8*(3), 294–299. https://doi.org/10.1016/j.jrras.2015.04.003

Ahmad, N., Rehman, J., ur Rehman, J., & Nasar, G. (2019). Assessments of 226Ra and 222Rn concentration in well and tap water from Sik, Malaysia, and consequent dose estimates. *Human and Ecological Risk Assessment*, *25*(7), 1697–1706. https://doi.org/10.1080/10807039.2018.1559034

Ahmed, K. M., & Burgess, W. G. (2003). Surface water and groundwater interaction in Bangladesh hydrogeology. *Groundwater Resources and Development in Bangladeshbackground to the Arsenic Crisis, Agricultural Potential and the Environment, AA Rahman, P. Ravenscroft, et Al., Bangladesh Centre for Advanced Studies, University Press Ltd., Dhaka, Bangladesh.*

Akar Tarim, U., Gurler, O., Akkaya, G., Kilic, N., Yalcin, S., Kaynak, G., & Gundogdu, O. (2012). Evaluation of radon concentration in well and tap waters in Bursa, Turkey. *Radiation Protection Dosimetry*, *150*(2), 207–212. https://doi.org/10.1093/rpd/ncr394

Al Zabadi, H., Musmar, S., Issa, S., Dwaikat, N., & Saffarini, G. (2012a). Exposure assessment of radon in the drinking water supplies: A descriptive study in Palestine. *BMC Research Notes*, *5*. https://doi.org/10.1186/1756-0500-5-29

Al Zabadi, H., Musmar, S., Issa, S., Dwaikat, N., & Saffarini, G. (2012b). Exposure assessment of radon in the drinking water supplies: A descriptive study in Palestine. *BMC Research Notes*, *5*. https://doi.org/10.1186/1756-0500-5-29

Bangladesh Bereau of Statistics. (2015). Poulation projection of Bangladesh- Dynamics and Trends. In *Journal of Chemical Information and Modeling* (Vol. 53, Issue 9).

Bhuiyan, M. A. H., Dampare, S. B., Islam, M. A., & Suzuki, S. (2015). Source apportionment and pollution evaluation of heavy metals in water and sediments of Buriganga River, Bangladesh, using multivariate analysis and pollution evaluation indices. *Environmental Monitoring and Assessment*, *187*(1). https://doi.org/10.1007/s10661-014-4075-0

Bodrud-Doza, M., Islam, S. M. D. U., Rume, T., Quraishi, S. B., Rahman, M. S., & Bhuiyan,

M. A. H. (2020). Groundwater quality and human health risk assessment for safe and sustainable water supply of Dhaka City dwellers in Bangladesh. *Groundwater for Sustainable Development*, *10*, 100374. https://doi.org/10.1016/J.GSD.2020.100374

Burgess, W. G., Hasan, M. K., Rihani, E., Ahmed, K. M., Hoque, M. A., & Darling, W. G. (2011). Groundwater quality trends in the dupi tila aquifer of Dhaka, Bangladesh: Sources of contamination evaluated using modelling and environmental isotopes. *International Journal of Urban Sustainable Development*, *3*(1), 56–76. https://doi.org/10.1080/19463138.2011.554662

Buriganga Riverkeeper : » History. (n.d.). Retrieved April 11, 2022, from https://burigangariverkeeper.org/history/

Copes, R., & Peterson, E. (2014). *Indoor Radon a Public Health Perspective*.

Darabi Fard, Z., Rahimi, M., Malakootian, M., & Javid, N. (2020). Studying radon concentration in drinking water resources in Zarand city (Iran) and its villages. *Journal of Radioanalytical and Nuclear Chemistry*, *326*(1), 33–39. https://doi.org/10.1007/s10967-020-07349-5

Dhaka, Geology - Banglapedia. (n.d.). Retrieved April 6, 2022, from https://en.banglapedia.org/index.php/Dhaka,_Geology

DWASA, I. W. M. (2008). Resource assessment and monitoring of water supply sources for Dhaka city. *Dhaka Water Supply and Sewerage Authority (DWASA), Dhaka*.

Faweya, E. B., Agbetuyi, O. A., Talabi, A. O., Adewumi, T., & Faweya, O. (2021). Radiological Implication of 222Rn Concentrations in Waters from Quarries Environs, Correlation with 226Ra Concentrations and Rocks Geochemistry. *Arabian Journal of Geosciences*, *14*(11). https://doi.org/10.1007/s12517-021-07385-9

Hasan, M K, Burgess, W. G., & Dottridge, J. (1998). Hydraulic and hydrochemical implications of river-aquifer interaction, Dhaka, Bangladesh. *Gambling with Groundwater-Physical, Chemical and Biological Aspects of Aquifer-Stream Relations*, 413–418.

Hasan, M Kamrul, Burgess, W., & Dottridge, J. (1999). The vulnerability of the Dupi Tila aquifer of Dhaka, Bangladesh. *IAHS-AISH Publication*, *259*, 91–98. https://www.researchgate.net/publication/228432423

Hoque, Mohammad A, Hoque, M. M., & Ahmed, K. M. (2007). Declining groundwater level and aquifer dewatering in Dhaka metropolitan area, Bangladesh: Causes and quantification. *Hydrogeology Journal*, *15*(8), 1523–1534. https://doi.org/10.1007/s10040-007-0226-5

Hoque, Mohammad Abdul. (2004). Hydrostratigraphy and Aqnifer Piezornetry of Dhaka City. *Institute of Water and Flood Management*. http://lib.buet.ac.bd:8080/xmlui/handle/123456789/1970

Hossain, S., Hasan Anik, A., Khan, R., Farah, ·, Ahmed, T., Abu, M., Siddique, B., Abdul, ·, Al, H., Khan, N., Saha, N., Abubakr, ·, Idris, M., & Alam, M. (n.d.). Public Health Vulnerability Due to the Exposure of Dissolved Metal(oid)s in Tap Water from a Mega City (Dhaka, Bangladesh): Source and Quality Appraisals. *Exposure and Health*. https://doi.org/10.1007/s12403-021-00446-0

International Atomic Energy Agency. (2019). Guidelines on soil and vegetation sampling for radiological monitoring. *Technical Reports Series No. 486*, *486*.

Islam, M., Van Camp, M., Hossain, D., Mizanur, ; Md, Sarker, R., Abdul, M., Bhuiyan, Q., & Walraevens, K. (2021). *Inferring major ion chemistry and quality of groundwater in Dupi Tila aquifer, Dhaka city, Bangladesh*.

Ismail, N. F., Hashim, S., Mohd Sanusi, M. S., Abdul Rahman, A. T., & Bradley, D. A. (2021a). Radon levels of water sources in the southwest coastal region of Peninsular Malaysia. *Applied Sciences (Switzerland)*, *11*(15). https://doi.org/10.3390/app11156842

Ismail, N. F., Hashim, S., Mohd Sanusi, M. S., Abdul Rahman, A. T., & Bradley, D. A. (2021b). Radon levels of water sources in the southwest coastal region of Peninsular Malaysia. *Applied Sciences (Switzerland)*, *11*(15). https://doi.org/10.3390/app11156842

Kareem, D. O., Ibrahim, A. A., & Ibrahiem, O. S. (2020). Heavy metal and radon gas concentration levels in Khasa River in Kirkuk City (NE Iraq) and the associated health effects. *Arabian Journal of Geosciences*, *13*(19). https://doi.org/10.1007/s12517-020-06037-8

Karim, S., Khatun, M., Ali, R. M. E., & Munsura Akther, K. (2019). Geomorphology and Geology of the Dhaka City Corporation Area-an Approach of Remote Sensing and GIS Technique. *International Journal of Astronomy*, *6*(2), 7–16.

http://www.openscienceonline.com/journal/aass

Lawal, W. (2021). Assessment of Annual Effective Dose Associated with Radon in Drinking Water from Gold and Bismuth Mining area of Edu, Kwara, North-central Nigeria. *Pollution*, *7*(1), 231–240. https://doi.org/10.22059/poll.2020.309470.892

Mittal, S., Rani, A., & Mehra, R. (2016). Radon levels in drinking water and soil samples of Jodhpur and Nagaur districts of Rajasthan, India. *Applied Radiation and Isotopes*, *113*, 53–59. https://doi.org/10.1016/j.apradiso.2016.04.017

Moldovan, M., Niţă, D. C., Cucos-dinu, A., Dicu, T., Bican-brişan, N., & Cosma, C. (2014). Radon concentration in drinking water and supplementary exposure in Băiţa-ŞTei Mining area, Bihor county (Romania). *Radiation Protection Dosimetry*, *158*(4), 447–452. https://doi.org/10.1093/rpd/nct258

Mustapha, A. O., Patel, J. P., & Rathore, I. V. S. (2002). Preliminary report on radon concentration in drinking water and indoor air in Kenya. *Environmental Geochemistry and Health*, *24*(4), 387–396. https://doi.org/10.1023/A:1020550103471

Nazir, S., Simnani, S., Sahoo, B. K., Mishra, R., Sharma, T., & Masood, S. (2020). Monitoring geothermal springs and groundwater of Pir Panjal, Jammu and Kashmir, for radon contamination. *Journal of Radioanalytical and Nuclear Chemistry*, *326*(3), 1915–1923. https://doi.org/10.1007/s10967-020-07451-8

Nita, D. C., Moldovan, M., Sferle, T., Ona, V. D., & Burghele, B. D. (2013). Radon concentrations in water and indoor air in North - West regions of Romania. *Romanian Reports of Physics*, *58*(SUPPL.), 2016–2020.

Öge, Ö., & Gökçe, H. (2018). Author ' s Accepted Manuscript. *Applied Radiation and Isotopes*. https://doi.org/10.1016/j.apradiso.2018.05.027

Orosun, M. M., Ajibola, T. B., Akinyose, F. C., Osanyinlusi, O., Afolayan, O. D., & Mahmud, M. O. (2021). Assessment of ambient gamma radiation dose and annual effective dose associated with radon in drinking water from gold and lead mining area of Moro, North-Central Nigeria. *Journal of Radioanalytical and Nuclear Chemistry*, *328*(1), 129–136. https://doi.org/10.1007/s10967-021-07644-9

Rahman, M. A., Wiegand, B. A., Badruzzaman, A. B. M., & Ptak, T. (2013). Analyse hydrogéologique de l'Aquifère Dupi Tila Supérieur, en vue de la mise en œuvre d'un

projet de recharge contrôlée de l’aquifère dans la ville de Dhaka, Bangladesh. *Hydrogeology Journal*, *21*(5), 1071–1089. https://doi.org/10.1007/s10040-013-0978-z

Rajashekara, K. M., Narayana, Y., & Siddappa, K. (2007). 222Rn concentration in ground water and river water of coastal Karnataka. *Radiation Measurements*, *42*(3), 472–478. https://doi.org/10.1016/j.radmeas.2006.12.010

Rani, S., Kansal, S., Singla, A. K., & Mehra, R. (2021). Radiological risk assessment to the public due to the presence of radon in water of Barnala district, Punjab, India. *Environmental Geochemistry and Health*, *43*(12), 5011–5024. https://doi.org/10.1007/s10653-021-01012-y

Şahin Bal, S., Tanrıverdi, E., Yalçın, S., Doğru, M., & Özbey, F. (2021). The radon concentrations of some waters in Bitlis (Turkey) and their dose estimates. *Environment, Development and Sustainability*, *23*(12), 17650–17667. https://doi.org/10.1007/s10668-021-01404-1

Salih, N. F. (2021). Determine the Contaminations of Radon in the Drinking Water Using NTDs (CR-39) and RAD7 Detectors. *Arabian Journal for Science and Engineering*, *46*(6), 6061–6074. https://doi.org/10.1007/s13369-020-05267-y

Shamsaddini, M., Negarestani, A., Malakootian, M., & Javid, N. (2020). Study of radon concentration of drinking water sources in adjacent areas of Sabzevaran fault. *Journal of Radioanalytical and Nuclear Chemistry*, *326*(2), 1437–1446. https://doi.org/10.1007/s10967-020-07426-9

Shivanandappa, K. C., & Yerol, N. (2018). Radon concentration in water, soil and sediment of Hemavathi River environments. *Indoor and Built Environment*, *27*(5), 587–596. https://doi.org/10.1177/1420326X16688522

States, U. (1999). RADON IN DRINKING WATER : Questions and Answers. *Environmental Protection*, *4607*, 1–4.

Thumvijit, T., Chanyotha, S., Sriburee, S., Hongsriti, P., Tapanya, M., Kranrod, C., & Tokonami, S. (2020a). Identifying indoor radon sources in Pa Miang, Chiang Mai, Thailand. *Scientific Reports*, *10*(1), 1–14. https://doi.org/10.1038/s41598-020-74721-6

Thumvijit, T., Chanyotha, S., Sriburee, S., Hongsriti, P., Tapanya, M., Kranrod, C., & Tokonami, S. (2020b). Identifying indoor radon sources in Pa Miang, Chiang Mai,

Thailand. *Scientific Reports*, *10*(1), 1–14. https://doi.org/10.1038/s41598-020-74721-6

UNSCEAR. (2010). *SOURCES AND EFFECTS OF IONIZING RADIATION United Nations Scientific Committee on the Effects of Atomic Radiation: Vol. I* (Issue c).

Urban Water Blueprint - Dhaka. (n.d.). Retrieved January 8, 2022, from http://water.nature.org/waterblueprint/city/dhaka/#/c=8:24.25758:90.26042

US EPA. (2012). *Radon in Drinking Water Regulations*.

Whitehead, P. G., Bussi, G., Peters, R., Hossain, M. A., Softley, L., Shawal, S., Jin, L., Rampley, C. P. N., Holdship, P., Hope, R., & Alabaster, G. (2019). Modelling heavy metals in the Buriganga River System, Dhaka, Bangladesh: Impacts of tannery pollution control. *Science of the Total Environment*, *697*, 134090. https://doi.org/10.1016/j.scitotenv.2019.134090

WHO. (2008a). *Guidelines for Drinking-water Quality, Third Edition*. *1*.

WHO. (2008b).

WHO. (2017). *Guidelines for drinking-water quality, 4th edition, incorporating the 1st addendum*.

Yashaswini, T., Ningappa, C., Niranjan, R. S., & Sannappa, J. (2020). Radon Concentration Level in Ground and Drinking Water around Kabini River Basin, Karnataka. *Journal of the Geological Society of India*, *95*(3), 273–278. https://doi.org/10.1007/s12594-020-1425-0

Yong, J., Liu, Q., Wu, B., Hu, Y., & Feng, G. (2021). Assessment of radiation dose hazards caused by radon and its progenies in tap water by the human dosimetric model. *Journal of Water and Health*, *00*(0), 1–13. https://doi.org/10.2166/wh.2021.113

Statements and Declarations

Funding

The authors declare that no funds, grants, or other support were received during the preparation of this manuscript.

Competing Interests

The authors have no relevant financial or non-financial interests to disclose.

Author Contributions

All authors contributed to the study conception and design. Material preparation, data collection and analysis were performed by [M.M. Mahfuz Siraz], [Mohammad Shafiqul Alam], and [Jubair Al Mahmud]. The first draft of the manuscript was written by [Mohammad Shafiqul Alam] and [Sudeb Chandra Das] and all authors commented on previous versions of the manuscript. [Selina Yeasmin] and [Dr. Afroza Shelley] supervised the research. [Dr. Mayeen Uddin Khandaker], [Dr. Shinji Tokonami], and [Dr. D.A. Bradley] checked the overall manuscript. All authors read and approved the final manuscript.

Data Availability

The datasets generated during and/or analyzed during the current study are not publicly available but are available from the corresponding author on reasonable request.

Ethics approval

This is an observational study. The Atomic Energy Centre Dhaka Research Ethics Committee has confirmed that no ethical approval is required.

Figure Click here to access/download;Figure;Plos One Figures.docx

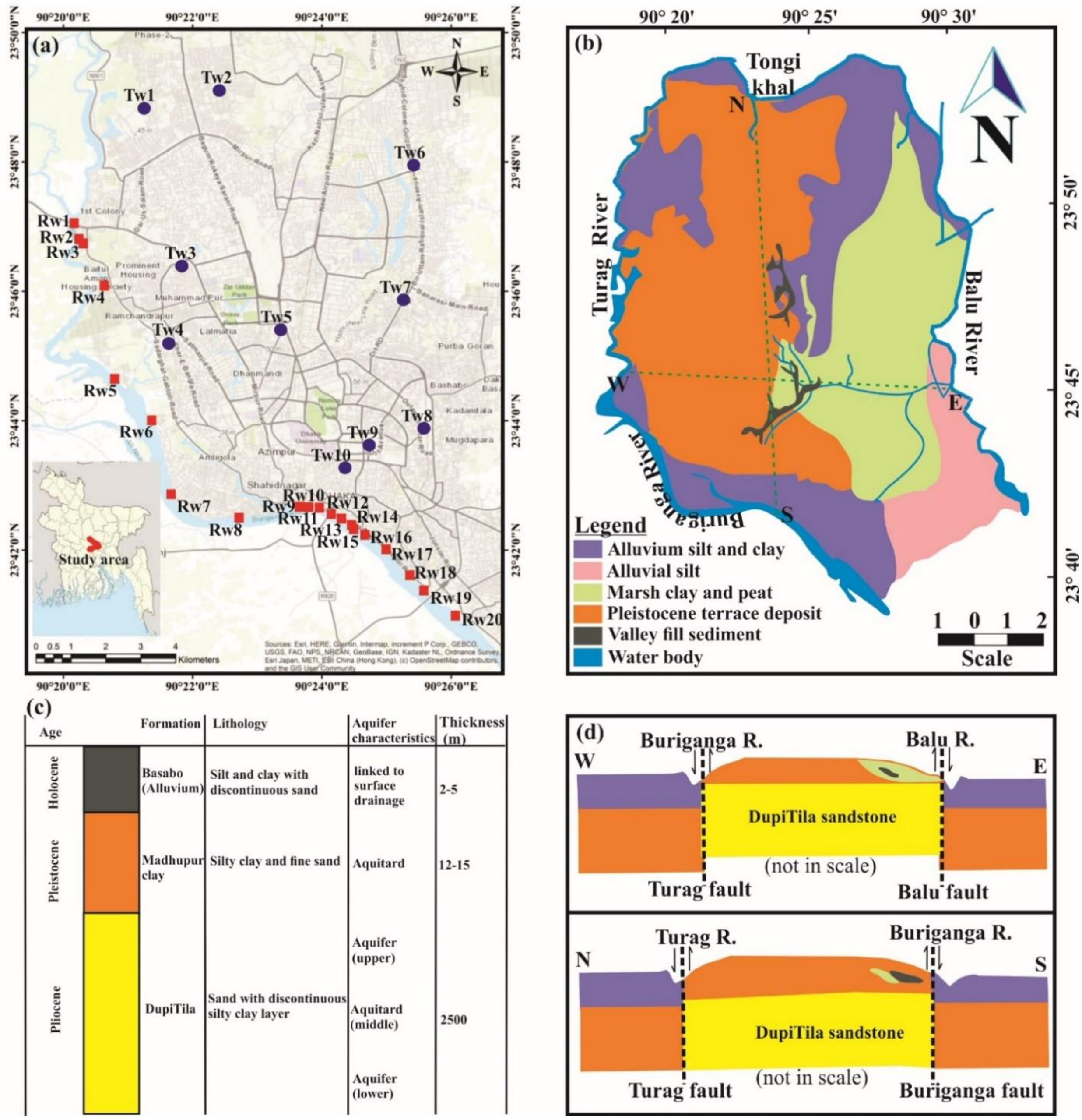

Fig 1. (a) The study area map showing the sampling location of river water (RW) and tap water (TW). (b) The geology of the study area. (c) Lithostratigraphy of the study area (Burgess et al., 2011). (d) Cross-section along the NS and EW direction in the study area.

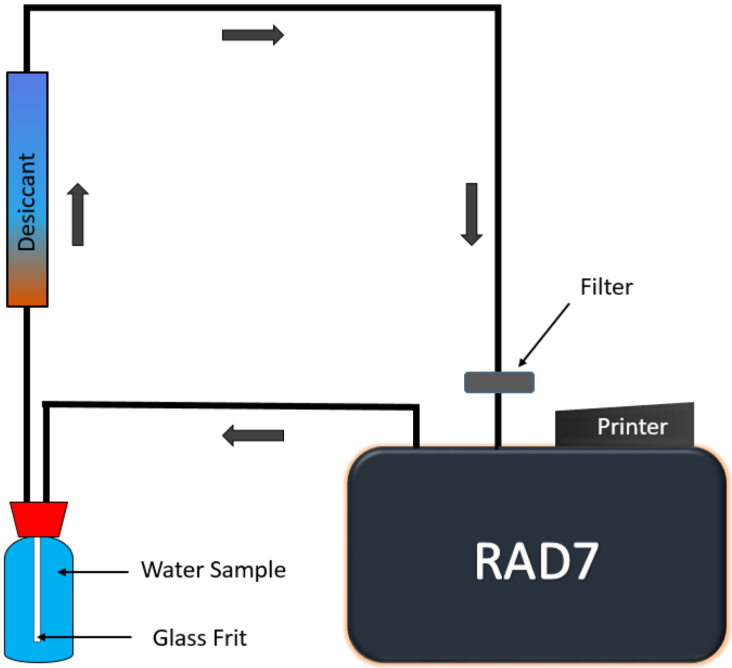

Fig 2. Schematic diagram of RAD-H_2O detector closed loop aeration system, where the air and water volume remain constant and independent of the flow rate.
