# Supplementary material for: A study on measuring the 222Rn in the Buriganga River and tap water of the megacity Dhaka
Source: PLoS One. 2023 May 23;18(5):e0286267. doi: 10.1371/journal.pone.0286267 (PMC10204947; doi:10.1371/journal.pone.0286267)
Supplement: Attachment — Submitted filename: Review comments_20221201.docx [file pone.0286267.s002.docx]

1) Title: should be modified following the points:

-‘radon, silent killer in Dhaka city’; based on the findings of this research work, radon has no potential risks for Dhaka city water. Thus, the issue about the silent killer in the title should be modified. In addition, this wording might be alarming for the public or government.

-pioneering study: there are already published articles for radon in tap water in Dhaka city

For example, (<http://article.scholarena.com/A-Study-of-Radon-Concentration-in-Tap-Water-of-Dhaka-City-Bangladesh.pdf>), therefore, the clarifying pioneering study should be reconsidered.

2) Abstract:

-authors clarified ‘this study poses a great significance for the radiological safety of public health’; however, the authors did not indicate any potential radiological risks/reasons for assessment of radon in river water at Dhaka city as background. It would be better if the authors clarified a few radiological exposures pathways/reasons- why radon in river water in Dhaka city is important shortly in the abstract.

-‘RAD H2O detector’; is not a detector, it is an accessory of RAD7.

-about the concentration of 1.537 ± 0.380 Bq/L and 0.675 ± 0.285Bq/L; what is the effective digit to be considered about the values? Why are three digits selected? It would be better to choose a logical concentration value throughout the paper.

-what is MCL? Nothing is clarified in the abstract.

-It would be better if the authors clarify the reason-why it is important to set a national safety limit for radon in Bangladesh. There is no potential risk in Bangladesh according to this article.

3)	Introduction:

-overall, the description of radon, its limits, characterization etc., has been mentioned much more than the information regarding the importance/background of radon study in water in Dhaka.

-‘emanates from the water’; radon emanation is a suitable word for soil/materials having grain as of radon generation process. For using it on water, it should be rechecked.

-‘safe water is essential for human lives, it is a matter of great concern to evaluate the radon level in the water’; as of the water quality/safety; radon might not a direct contaminant (following EPA)
 <https://www.epa.gov/dwreginfo/drinking-water-regulations>

<https://www.epa.gov/ground-water-and-drinking-water/national-primary-drinking-water-regulations#Radionuclides>

<https://nepis.epa.gov/Exe/ZyPDF.cgi?Dockey=30006644.txt>

-It is better to use either ^222^Rn or radon; one form throughout the texts.

-‘Almost every developed country has its national reference limit of radon in water’; this information should be checked; please insert reliable references to insert this information.

- The authors clarified, ‘’no studies on radon measurement in surface water, tap water, or indoor air’’ ; there are many published articles dealing with radon in bangladesh-

<https://doi.org/10.1093/oxfordjournals.rpd.a082065>

<https://doi.org/10.1016/0969-8078(93)90079-J>

<https://doi.org/10.1016/j.radmeas.2008.03.050>

<https://doi.org/10.1016/1359-0189(91)90029-H>

<https://doi.org/10.1016/1359-0189(88)90200-2>

<https://link.springer.com/article/10.1007/s10661-019-7650-6>

<http://article.scholarena.com/A-Study-of-Radon-Concentration-in-Tap-Water-of-Dhaka-City-Bangladesh.pdf>

It is recommended to look at the papers and insert associated references to understand the importance of radon research in Bangladesh/Dhaka city.

-Buriganga refers to 20% of the total water demand of Dhaka city dwellers; please insert a suitable reference here.

4) Methodology:

-extensive discussion about the Geology of Dhaka city was nicely given. However, sufficient information is not mentioned in the methodology or results section about the potential radiological risks, relationship with radionuclide distribution, or radon exposure following Geology. Please insert some points on radiological risks based on the Geology.

-sample collection through plastic bottle: please specify what criteria were filled up collecting water samples to avoid escaping radon. What type of plastic bottle was used, and what arrangements were taken for the bottle cap? It is recommended to use glass bottles; it is relatively easier to escape radon from different plastic bottles. IAEA materials explained such precautions for collecting water-

<https://www.iaea.org/sites/default/files/19/11/radon-presentation-bochicchio.pdf>

-please specify your assessed lower detection limit of RAD7 in measuring water in the research, as the results exhibit lower values.

-dosimetry calculation: why CW=730 L/day is used? *Thumvijit et al., 2020* specified 2L/day.

5) Results in Discussion:

-why is the effective dose for radon (both river and tape water) achieved from ingestion and inhalation exhibited around similar levels? Studies from other countries (cited in this article) possess much differences between the two doses. It would be very interesting if the authors interpret the reasons.

-please specify how this study is consistent with studies from other countries.

-why ‘a radon mitigation treatment plant should be introduced’ with no elevated radon levels?

6) Reference: It is necessary to verify all the references cited in the text and the reference list needs to be standardized->

-authors names were written two times in one reference. Moreover, authors names could not be found in the website (see reference Ahmed, K. M., & Burgess, W. G. (2003)).

-same reference was written as a and b. (Al Zabadi, H., Musmar, S., Issa, S., Dwaikat, N., & Saffarini, G. (2012a). It should be one reference.

-reference could not be found on the journal website with the correct content. In cited *Journal of Chemical Information and Modeling (Vol. 53, Issue 9)*, nothing is mentioned about the Bangladeshi population on the website.

-incorrect formatting(n.d) was frequently used: Buriganga Riverkeeper : » History. (n.d.)

-missing title; only journal name and author name were included (Copes, R., & Peterson, E. (2014). Indoor Radon a Public Health Perspective).

-please check if it is scientifically ok with some references as suitable for scientific journals (i.e.,Banglapedia).

-ResearchGate should not be a reference source for journal articles to be cited.

7) English language: the overall English language should be improved. There are problems with wording and grammar.

Comments on scientific content:

- scientific problem-oriented background has not been described in the text especially in the introduction part? What are the scientific purposes to be newly understood in this work?
- as for the first time obtained data, it is important to ascertain the radon level in water in Dhaka city river. However, it would be better to make some statistical analysis to determine how the obtained data from two sources or sample collection points are significant and associated with each other.
- how to choose sampling areas/positions/sapling numbers? How to decide the river and tap water in this study for dose calculation?
- In conclusion part, considering this work’s purpose, it is important to insert the new findings and recommendations for future research as an original research articles (as this is one of the preliminary studies for radon in water).
