# Supplementary material for: A study on measuring the 222Rn in the Buriganga River and tap water of the megacity Dhaka
Source: PLoS One. 2023 May 23;18(5):e0286267. doi: 10.1371/journal.pone.0286267 (PMC10204947; doi:10.1371/journal.pone.0286267)
Supplement: Attachment — Submitted filename: Response to Reviewer.docx [file pone.0286267.s004.docx]

**Response to academic editor and reviewer(s)**

Respected Academic Editor and Reviewers,

Thank you so much for your comments. We are very grateful to you for the time and intelligence that you have shared with us. It is a great learning opportunity for us through these reviews. Our responses to the comments are highlighted in red color in the revised manuscript.

Comments and Suggestions for Authors:

**Reviewer #3 comment:** The manuscript has been updated nicely. However, a few points below are still necessary as minor corrections:

**Reviewer 3, comment 1:** Although a new paragraph regarding the ‘Radiological risks based on geology’ has been added; the impact on the research area of Dhaka city (also surrounding areas) seemed to be missing. It would be better if some discussion on geology and Dhaka city is given specifically in the text.

**Response:** Thank you very much for your suggestion. As per your suggestions, we have taken the section 2.3 ‘Radiological risks based on geology’ in the result and discussion part under the section “3.3 Radiological risks based on geology of the study area”, (page no. 15, line no. 318-353), where the possible impact of radon is discussed based on the geology of Dhaka city and its periphery region.

**Reviewer 3, comment 2:** In the dose calculation equation, CW (daily water consumption) = 3 L/day is used. How suitable is it based on Dhaka city/Bangladeshi people’s lifestyle?

**Response:** We thank the reviewer for this valuable comment. We have added a citation about the daily water consumption of Bangladeshi people. (page no. 08, line no. 213).

**Reviewer 3, comment 3:** Scientific purposes are still not adequately discussed. The conclusion part should introduce and focus on the new findings. Recommendations given in the conclusion part are not based on the findings of this research; they are too general. In this stage, this document would be Review Paper, not Original Paper.

**Response:** We thank the reviewer for the opportunity to clarify this issue. We have added the following lines in the results section (page no.15-16, line no.354-369), “The Stochastic radiation model is based on the probabilistic nature of radiation-induced cancer and suggests that there is no threshold limit for radiation exposure below which the risk of cancer becomes zero. This means that even a single atom of ^222^Rn in water can potentially cause severe damage to the body by ionizing molecules and damaging cellular structures. Therefore, it is important to closely monitor the levels of ^222^Rn in water, as even low concentrations can pose a risk to human health. Despite all measured values of ^222^Rn levels in the tap and river water of Dhaka being below the limit set by the USEPA and WHO, continuous monitoring is essential to ensure that the levels remain within safe limits. The USEPA limit for ^222^Rn in drinking water is 11.1 Bq/L, while the WHO guideline value is 100 Bq/L. In this study, the measured levels of ^222^Rn in tap water and river water ranged from 0.56 ± 0.30 to 3.06 ± 0.61 Bq/L and from 0.35 ± 0.18 to 1.16 ± 0.61 Bq/L, respectively. The corresponding effective doses were found to be below the limit of 0.1 mSv/y recommended by the WHO [8]. Nevertheless, given the potential health risks associated with even small amounts of ^222^Rn in water, continuous monitoring of its levels is essential to ensure public safety. This study on ^222^Rn levels in Dhaka's water may provide valuable insights for future research on radiation exposure and human health.”

We have added the following lines in the recommendation section (page no. 17, line no. 400-414)

“A few recommendations are proposed for future ^222^Rn-related works,

- Expansion of the study to other regions of Bangladesh to get a better understanding of the national distribution of ^222^Rn levels in different dwelling media, including water, air, and soil is essential.
- It is necessary to investigate the potential impact of local geological factors, such as soil type and groundwater composition, on the levels of ^222^Rn in water sources.
- Exploration of the relationship between ^222^Rn exposure and cancer incidence rates in the region is crucial to gain a better understanding of the public health implications of 222Rn exposure in Dhaka and other regions of Bangladesh.
- A risk assessment study should be conducted to evaluate the potential health risks associated with chronic exposure to low levels of ^222^Rn in water sources in Dhaka and other regions of Bangladesh.
- A long-term monitoring program needs to be developed and implemented to track changes in ^222^Rn levels over time and ensure that public health safety measures remain effective.”

**Reviewer 3, comment 4:** Comparison/association of tap water and river water for concentration and dose have not been sufficiently discussed from environmental/scientific viewpoints.

**Response:** We are grateful to the reviewer for this comment. We have already mentioned some information relevant to the environmental/scientific viewpoints in the introduction section (page no. 03, line no. 77-85). However, as per your suggestion, we have added the following sentences in the results and discussion section (page no. 16, line no. 369-389), “Many advanced countries have established national reference limits for radon in water and indoor air in order to ensure radiological safety and protect public health. However, Bangladesh currently lacks such a reference level for ^222^Rn in water, despite millions of people in the Dhaka megacity relying solely on tap water for daily household activities, including washing, bathing, drinking, and cooking. Given that the Buriganga river serves as a major transportation hub and facilitates to many businesses and trade centers, there is a greater likelihood of ^222^Rn exposure for the general population. In terms of concentration, it has been observed in this study that, tap water has a higher concentration of ^222^Rn than the river water. This is because, radon in river water can be easily diluted due to greater surface and interactions. However, this can vary depending on factors such as the geology of the area and the treatment processes used for tap water. When it comes to dose, the risk of exposure to ^222^Rn from tap water is greater than from river water, as people are likely consume more tap water than river water. However, exposure to ^222^Rn in river water can still occur through activities such as swimming and fishing. Overall, from an environmental and scientific viewpoint, it is important to measure and monitor the concentration of ^222^Rn in both tap water and river water to ensure that exposure levels do not exceed safe limits. This can help to protect public health and ensure that the water we use for daily activities is safe and free from harmful contaminants. Therefore, this study measures the ^222^Rn levels in both tap water and Buriganga river water to determine if they fall within the safe limits and ultimately ensure the radiological safety of the public. This study on ^222^Rn levels in Dhaka city's water may provide valuable insights for future research on radiation exposure and human health.”

**Reviewer 3, comment 5:** Lower detection limit has newly been clarified in the revised manuscript. However, that was based on other studies. It would be better to use the value using the detector, which was used in your research considering your measurement condition.

**Response:** We thank the reviewer for indicating this issue. The lower detection limit of our detector is consistent with the cited paper, that is why we cited that paper.
